# Delayed presentation of late-onset cerebrospinal fluid rhinorrhoea following dopamine agonist therapy for giant prolactinoma

**DOI:** 10.1530/EDM-14-0020

**Published:** 2014-11-01

**Authors:** J K Prague, C L Ward, O G Mustafa, B C Whitelaw, A King, N W Thomas, J Gilbert

**Affiliations:** 1 Department of Endocrinology, King's College Hospital, London, SE5 9RS, UK; 2 Department of Neurosurgery, King's College Hospital, London, SE5 9RS, UK; 3 Department of Clinical Neuropathology, King's College Hospital, London, SE5 9RS, UK

## Abstract

Therapeutic shrinkage of prolactinomas with dopamine agonists achieves clinical benefit but can expose fistulae that have arisen as a result of bony erosion of the sella floor and anterior skull base by the invasive tumour, resulting in the potential development of cerebrospinal fluid (CSF) rhinorrhoea, meningitis, and rarely pneumocephalus. Onset of symptoms is typically within 4 months of commencing therapy. The management is typically surgical repair via an endoscopic transnasal transsphenoidal approach. A 23-year-old man presented to the Emergency Department with acute left limb weakness and intermittent headaches. Visual fields were full to confrontation. Immediate computed tomography and subsequent magnetic resonance imaging (MRI), demonstrated a 5 cm lobular/cystic mass invading the right cavernous sinus, displacing and compressing the midbrain, with destruction of the bony sella. He was referred to the regional pituitary multidisciplinary team (MDT). Serum prolactin was 159 455 mIU/l (7514.37 ng/ml) (normal ranges 100–410 mIU/l (4.72–19.34 ng/ml)). Cabergoline was commenced causing dramatic reduction in tumour size and resolution of neurological symptoms. Further dose titrations were required as the prolactin level plateaued and significant residual tumour remained. After 13 months of treatment, he developed continuous daily rhinorrhea, and on presenting to his general practitioner was referred to an otolaryngologist. When next seen in the routine regional pituitary clinic six-months later he was admitted for urgent surgical repair. Histology confirmed a prolactinoma with a low proliferation index of 2% (Ki-67 antibody). In view of partial cabergoline resistance he completed a course of conventional radiotherapy. Nine months after treatment the serum prolactin had fallen to 621 mIU/l, and 12 months after an MRI showed reduced tumour volume.

**Learning points:**

- CSF rhinorrhoea occurred 13 months after the initiation of cabergoline, suggesting a need for vigilance throughout therapy.
- Dedicated bony imaging should be reviewed early in the patient pathway to assess the potential risk of CSF rhinorrhoea after initiation of dopamine agonist therapy.
- There was a significant delay before this complication was brought to the attention of the regional pituitary MDT, with associated risk whilst left untreated. This demonstrates a need for patients and healthcare professionals to be educated about early recognition and management of this complication to facilitate timely and appropriate referral to the MDT for specialist advice and management. We changed our nurse-led patient education programme as a result of this case.
- An excellent therapeutic response was achieved with conventional radiotherapy after limited surgery having developed partial cabergoline resistance and CSF rhinorrhoea.

## Background

Prolactinomas are the most common subtype of pituitary tumours (57%), occurring particularly in women (1). However, giant prolactinomas are very rare tumours (4% of all prolactinomas in one series) (2), and have a significant male preponderance (3). A recent definition has been suggested as a pituitary adenoma with a diameter of 40 mm or more, significant extrasellar extension, very high prolactin concentrations and no concomitant growth hormone (GH) or adrenocorticotrophin (ACTH) secretion (4). Such large, often invasive, tumours typically present with neurological rather than endocrine symptoms (3), and pose a particular challenge to the pituitary multidisciplinary team (MDT). Giant prolactinomas typically respond rapidly to dopamine agonist therapy, but may require multimodal therapeutic options including surgery and radiotherapy to achieve normalisation of prolactin and tumour control (5). Surgery may also be required if acute complications develop such as cerebrospinal fluid (CSF) rhinorrhoea, apoplexy, or chiasmal herniation. Dopamine agonist resistance is more common in patients who develop CSF rhinorrhoea after medical treatment for giant prolactinoma (6).

Isolated case reports of CSF rhinorrhoea after standard medical treatment with dopamine agonists for prolactinomas are rare but appear in the literature (7). Therapeutic tumour shrinkage achieves clinical benefit but can expose fistulae that have arisen as a result of bony erosion of the sellar floor and anterior skull base by the invasive tumour. Onset of symptoms is typically within 4 months of commencing therapy (7) but can occur later: in the literature the longest period after initiation of treatment is 17 months (8). A recognised complication of CSF rhinorrhoea is meningitis, which has an associated annual risk of 10% (9), and rarely pneumocephalus. The management is typically surgical repair via an endoscopic transnasal transsphenoidal approach, and may involve the use of dural substitutes, autologous fat or fascial grafts, fibrin tissue glue, turbinate mucosal harvest, a pedicled or free nasoseptal flap or packing depending on the size and number of defects.

## Case presentation

A 23-year-old man presented to the Emergency Department with acute weakness in his left arm, dragging of his left leg whilst running and recent intermittent headaches. Examination revealed a partial right ptosis and inadequate androgenisation. Visual fields were full to confrontation. His only previous medical history was of delayed achievement of language milestones during childhood.

## Investigation

**Figure 1:**
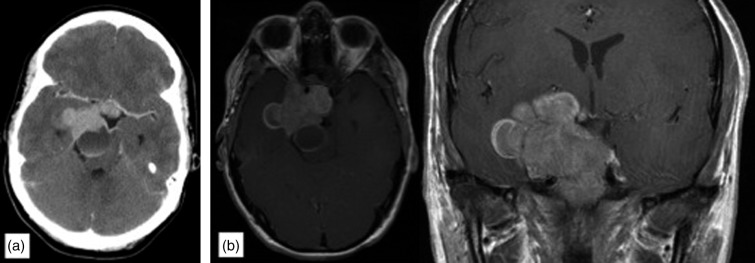
(a) Computed tomography of the head at presentation shows a large lobular/cystic mass invading the right cavernous sinus, displacing and compressing the midbrain. (b) Magnetic resonance image of the brain at presentation further characterises the mass (T1 sagittal and coronal).

Immediate computed tomography (CT) demonstrated a large solid and cystic mass, which was further characterised by magnetic resonance imaging (MRI) as a 5 cm lobular/cystic mass invading the right cavernous sinus, displacing and compressing the midbrain, with destruction of the bony sella (Fig. 1). He was referred to the regional pituitary MDT who advised a serum prolactin, which was 159 455 mIU/l (normal range 100–410 mIU/l) (to convert mIU/l to ng/ml divide by 21.2≡7514.37 ng/ml (normal range 4.72–19.34 ng/ml)) and a pituitary profile. Of note, he had a testosterone of 4.8 nmol/l (normal range 10–30 nmol/l) with gonadotrophins in the low-normal range (luteinizing hormone (LH) 2.1 IU/l (normal range 1.5–9.3 IU/l)) and follicle-stimulating hormone (FSH) 3.9 IU/l (normal range 1.8–10 IU/l)). Other anterior pituitary hormone results were as follows: free T_4_ 9.5 pmol/l (normal range 9–25 pmol/l), thyrotrophin (TSH) 1.6 mU/l (0.3–5.5 mIU/l), 1100 h cortisol 276 nmol/l (normal range 130–580 nmol/l), insulin-like growth factor 1 (IGF1) 189 μg/l (normal range 116–358 μg/l).

## Treatment

**Figure 2:**
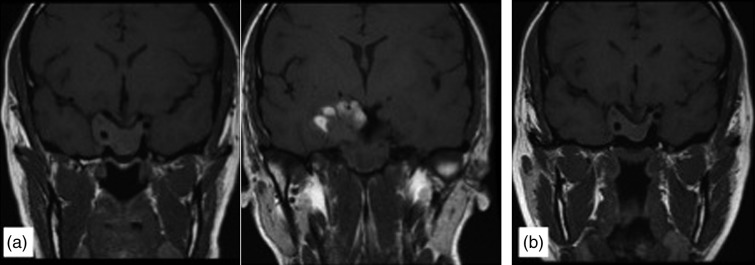
(a) Interval magnetic resonance images (MRI) after 3 months of cabergoline therapy shows dramatic reduction in the size of the tumour with decompression of the midbrain and some new haemorrhagic components. (b) Interval MRI after 10 months of cabergoline therapy shows continued reduction in tumour bulk but the rate of change is slower.

Cabergoline was commenced (initially 250 μg twice/week) causing a dramatic reduction in tumour size and resolution of his neurological symptoms. The prolactin level continued to fall with subsequent normalisation of his testosterone level (testosterone increased from 4.8 to 12.0 nmol/l after 3 months, and to 17.4 nmol/l after 7 months without replacement), and interval MRIs reviewed in the multidisciplinary pituitary meeting showed continued reduction in tumour bulk (Fig. 2), although repeated increments in the dose of cabergoline were required (up to 500 μg three times/week) because the rate of change slowed.

After 12 months of therapy, the prolactin level plateaued at 20 000 mIU/l (943.40 ng/ml) with significant remaining residual sellar and right parasellar tumour. Consequently, the cabergoline dose was increased to 500 μg/day, following which he developed continuous daily rhinorrhoea.

**Figure 3:**
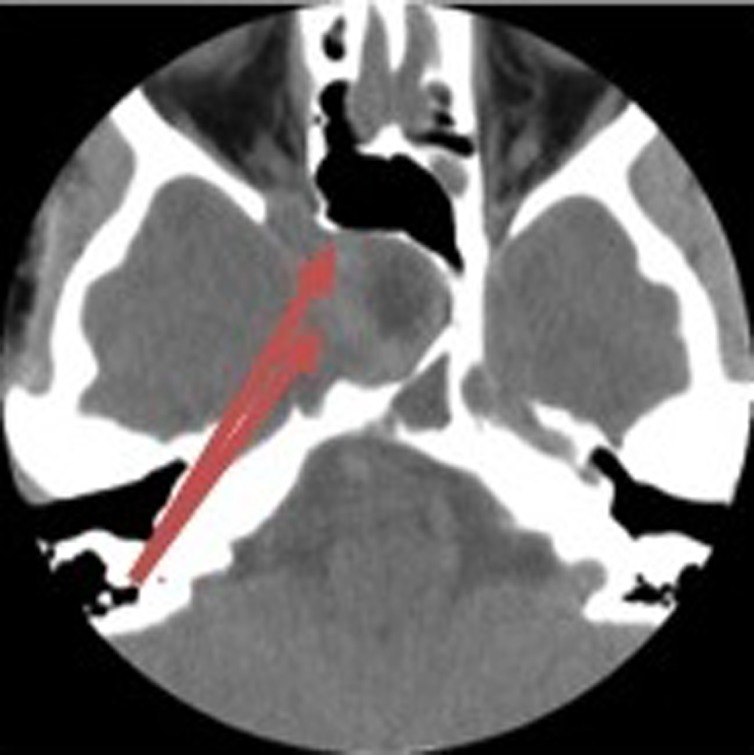
Computed tomography of the pituitary: arrows highlight areas of thinned and deficient bony sella.

The patient presented to his general practitioner (family doctor) complaining of nasal discharge, who referred him to an otolaryngology clinic after determining the glucose concentration of the clear fluid. The pituitary team was not informed and he continued on his current dose of cabergoline. When next seen in the routine regional endocrine clinic 6 months later, he was admitted for urgent surgical repair. CT pituitary confirmed the likely site of the leak was the left basisphenoid, where there was marked thinning of the bone (Fig. 3). Serum prolactin was 5410 mIU/l (255.19 ng/ml).

He proceeded to endoscopic transnasal transsphenoidal repair of the skull base defect and simultaneous biopsy. When the anterior sphenoid wall was taken down to expose the bony sella, an obvious defect was seen with tumour prolapsing into the sphenoid sinus. The surrounding mucosa was stripped to expose the bony defect, which was repaired under strict haemostasis with layered Fibrillar Surgicel^®^ (Ethicon, Norderstedt, Germany), a pedicled nasoseptal mucosal flap and Tisseel (Baxter, IL, USA). The sphenoid was carefully packed with Nasopore^®^ (Polyganics, Groningen, The Netherlands). Post-operatively he had no further rhinorrhoea and only transient mild diabetes insipidus. He continued on the same dose of cabergoline (500 μg/day).

**Figure 4:**
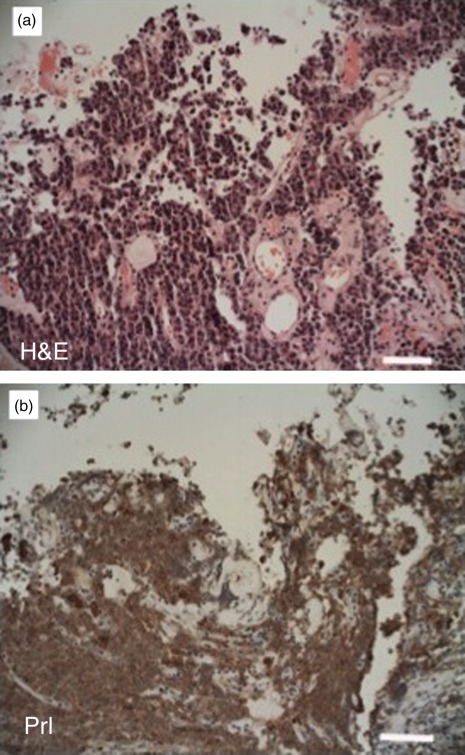
(a) Photomicrograph hematoxylin and eosin (H&E) stain (×20) scale bar represents 50 microns. (b) Photomicrograph prolactin (Prl) stain, scale bar represents 50 microns.

Histology confirmed a prolactinoma (Fig. 4) with a low proliferation index of 2% (using the Ki-67 antibody).

**Figure 5:**
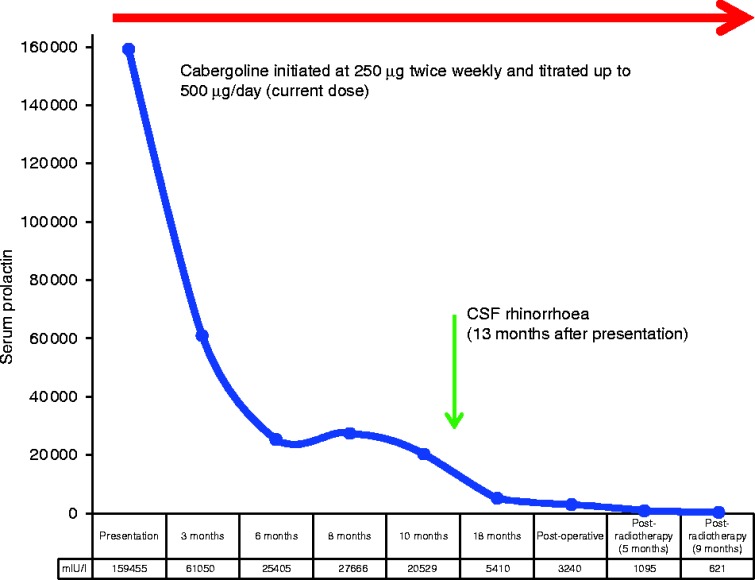
Serum prolactin (mIU/l) over time.

He represented on the 17th post-operative day with a 7-day history of new rhinorrhoea. Again, via an endoscopic transnasal transsphenoidal approach, the previous repair was examined, which revealed a breach in the infero-medial corner. The flap was taken down, the bony edges of the defect stripped further, a fat graft placed in the defect and the pedicled mucosal flap then replaced over the defect, which was layered with Fibrillar Surgicel^®^ (Ethicon) and Tisseel (Baxter). The sphenoid was carefully packed with Nasopore^®^ (Polyganics). His rhinorrhoea again resolved immediately post-operatively and he only had mild transient diabetes insipidus. Post-operative prolactin level was 3240 mIU/l (152.83 ng/ml) (Fig. 5).

In view of partial cabergoline resistance, he completed a course of conventional radiotherapy (45 Gy in 25 fractions), which was carefully considered as he had intact pituitary function and was yet to complete his educational studies at University.

## Outcome and follow-up

Nine months after radiotherapy, the serum prolactin had fallen to 621 mIU/l (29.29 ng/ml) (Fig. 5), and an MRI after 12 months showed a reduction in tumour volume. His CSF rhinorrhoea has not recurred in the 23 months of follow-up to date. He continues on the same dose of carbergoline (500 μg/day).

## Discussion

In this case, CSF rhinorrhoea occurred 13 months after the initiation of dopamine agonist therapy, suggesting a need for vigilance throughout the duration of treatment. Reports in the literature suggest the onset of symptoms to typically occur between 3 days and 4 months after starting medical therapy (7), although one report describes a case as late as 17 months after (8). There was a long delay before this complication was brought to the attention of the regional pituitary MDT, with significant associated risk whilst being left untreated. This suggests a need for patients and healthcare professionals to be educated about early recognition and appropriate management of this complication to facilitate timely and appropriate referral. Dedicated bony imaging would have also been helpful in predicting the likely development of CSF rhinorrhoea. Following this case, we revised both our protocol for investigating patients with macroprolactinoma to include early dedicated bone imaging to risk stratify for potential development of CSF leak, and our nurse-led patient education programme.

Despite initial dramatic response to dopamine agonist therapy with resolution of neurological symptoms as expected (4), the rate of change slowed and partial cabergoline resistance developed. Resistance to dopamine agonists likely reflects a change in the tumour biology with reduced gene expression of D2 receptors (10) and PRB3 (11) within the tumour cells rather than a response to, or effect of, therapy. However, dopamine agonist resistance is more common in giant prolactinomas with subesequent CSF rhinorrhoea (6), and the mechanism for this is not completely understood (5). Surgery was required to repair a bony defect causing CSF rhinorrhoea, and biopsy, rather than formal debulking. Radiotherapy was carefully considered in this case, particularly because anterior pituitary function was intact and the patient was yet to complete his educational studies. However, it appears that an excellent response was achieved: 9 months after conventional radiotherapy his prolactin had nearly normalised (621 mIU/l) and no loss of anterior pituitary function had occurred. Radiotherapy in addition to continued dopamine agonist therapy has previously been shown to aid long-term tumour control (12). There are very few studies that have examined the effect of radiotherapy in giant prolactinomas however, so it is difficult to determine whether the response to radiotherapy was as expected, but typically the tumour response to pituitary radiotherapy is slower, than in this case, with a 25–30% risk of anterior hormone deficiency that can occur for up to 19 years after treatment (13) (14). There has been some suggestion that partial tumour resection may improve sensitivity to dopamine agonist therapy, whereby improved responses can be achieved despite lower doses of cabergoline post-operatively (15). However, there has been no sub-group analysis for patients specifically with giant prolactinoma (15). In our reported case, the same pre-operative dose of cabergoline was also continued post-operatively and therefore this may explain some of the perceived clinical and biochemical improvement after surgery and radiotherapy.

## Patient consent

Written informed consent for publication has been obtained from the patient.

## Author contribution statement

J K Prague wrote the first draft and all authors contributed to the editing process. All authors were involved in the direct care of the patient.
